# Tight Junction in the Intestinal Epithelium: Its Association with Diseases and Regulation by Phytochemicals

**DOI:** 10.1155/2018/2645465

**Published:** 2018-12-16

**Authors:** Bonggi Lee, Kyoung Mi Moon, Choon Young Kim

**Affiliations:** ^1^Korean Medicine (KM)-Application Center, Korea Institute of Oriental Medicine (KIOM), 70 Cheomdan-ro, Dong-gu, Daegu 41062, Republic of Korea; ^2^Department of Food Science and Nutrition, Pukyong National University, 45 Yongso-ro, Nam-gu, Busan 48513, Republic of Korea; ^3^Department of Food and Nutrition, Yeungnam University, Gyeongsan, Gyeongbuk 38541, Republic of Korea

## Abstract

The intestine plays an essential role in integrating immunity and nutrient digestion and absorption. Adjacent intestinal epithelia form tight junctions (TJs) that are essential to the function of the physical intestinal barrier, regulating the paracellular movement of various substances including ions, solutes, and water across the intestinal epithelium. Studies have shown that TJ dysfunction is highly associated with metabolic and inflammatory diseases. Thus, molecular and nutritional factors that improve TJ activity have gained attention in the pharmaceutical and medicinal fields. This review focuses on the association between TJ and diverse pathological conditions, as well as various molecular and nutritional interventions designed to boost TJ integrity.

## 1. Introduction

**Figure 1:**
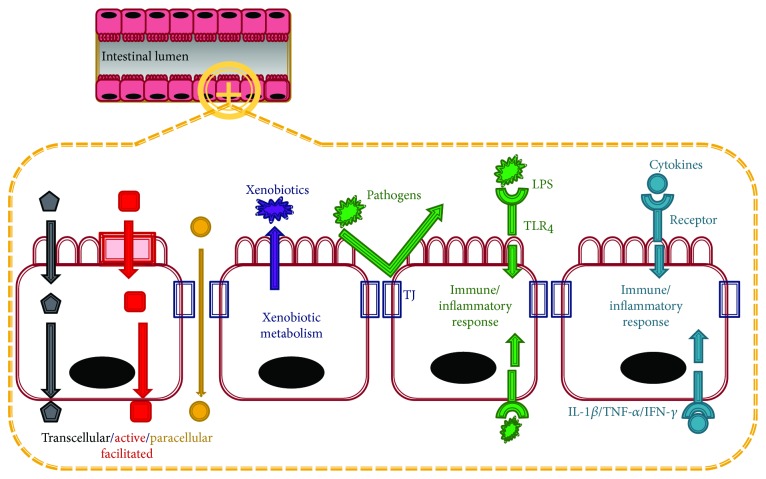
Functions of the small intestine. The small intestinal epithelium plays a role in the absorption of nutrients through transcellular (nutrients passing through the cells), facilitated and active (nutrients passing through the membrane via transport proteins), and paracellular (nutrients passing between the tight junction (TJ) between cells) transports. The small intestinal epithelium also performs barrier functions because of the presence of TJ and the xenobiotic detoxification system. TJ acts as a physical barrier to pathogens and large harmful molecules while enzymes involved in xenobiotic metabolism detoxify harmful compounds. Furthermore, intestinal cells expressing toll-like receptors (TLRs) and cytokine receptors respond to lipopolysaccharides (LPS) and proinflammatory cytokines (interleukin-1 beta, tumor necrosis factor-alpha, and interferon gamma) triggering intracellular signaling pathways.

The intestinal epithelium forms the lining of the small intestine. Each epithelium has a brush border, villi, crypt, and basolateral plasma membrane structure. The small intestine not only absorbs nutrients from the diet but also offers a physical barrier—assisted by the tight junctions (TJs) formed by neighboring epithelial cells—and a biological barrier, both of which act against extracellular substances such as microorganisms, antigens, and xenobiotics (Figure 1). Moreover, the small intestine secretes a wide range of hormones that regulate its internal functions as well as energy metabolism throughout the body.

The main function of the small intestine is the absorption of nutrients. The intestinal epithelium expresses digestive enzymes, transporters of specific nutrients, and metabolic enzymes. The small intestine also mediates signal transduction and produces bioactive compounds. The intestinal epithelium responds to various inflammatory and oxidative stresses induced by bacterial toxins, proinflammatory cytokines (TNF-*α* and IL-1*β*), and other components through various receptors, including toll-like receptors (TLRs) on the plasma membrane of the epithelium.

TJs contribute to the function of the physical intestinal barrier by regulating the paracellular movement of ions, solutes, and water across the intestinal epithelium, while the detoxification system contributes a biological barrier against xenobiotics. In addition, TJ integrity is related to the functions of the intestinal epithelium. Data from clinical trials and basic science suggest that the TJ barrier plays an essential role in the pathogenesis of systemic and intestinal disorders. Therefore, this review will discuss the association between TJ and various metabolic and inflammatory diseases, as well as the molecular and nutritional controls of intestinal TJ.

## 2. Tight Junction and Tight Junction-Associated Proteins

**Figure 2:**
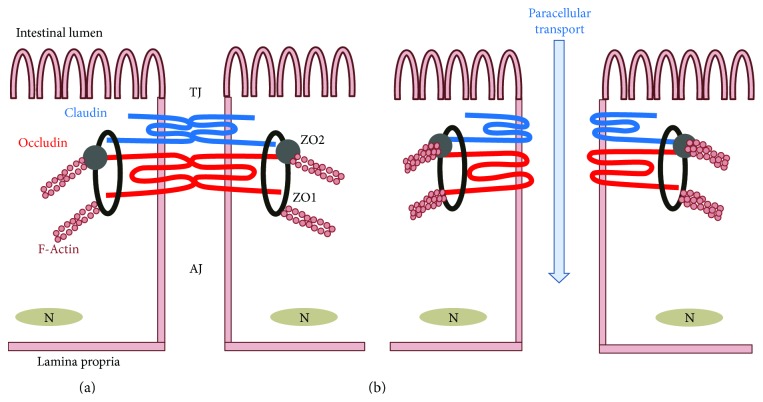
Structure of intestinal epithelial tight junction (TJ) and TJ-related proteins. (a) Assembly of TJ-related proteins including claudin, occludin, ZO-1 and -2, and F-actin forms a TJ structure, which confers physical barrier function to the small intestine. (b) Upon luminal stimulation, the TJ opens, leading to paracellular transport of extracellular components.

TJ is generated by the assembly of multiple proteins located near the apical part of the epithelium between neighboring cells (Figure 2) and controls the permeability of the paracellular transport pathway. TJ plays a pivotal role in maintaining intestinal barrier function and consists of two functional protein categories, integral transmembrane proteins that form a network between adjacent cell membranes and peripheral membrane or plaque proteins. TJ integrity is dynamically regulated by the arrangement of actin and the interaction between integral transmembrane and peripheral membrane proteins. Four integral transmembrane proteins are occludin, claudins, junctional adhesion molecule (JAM), and tricellulin. Peripheral membrane adaptor proteins zonula occludens-1 (ZO-1), ZO-2, and ZO-3 act as bridges to connect integral membrane proteins to the actin cytoskeleton and to other signaling proteins. The phosphorylation, distribution, and expression levels of TJ proteins play a critical role in regulating TJ barrier function. They are tightly regulated by the following intracellular signal transduction pathways: protein kinase C (PKC), A (PKA), and G (PKG) signalings; phosphatase, Rho, myosin light chain (MLC) kinase (MLCK), and MAPK signaling; and the PI3K/Akt pathway [1, 2].

### 2.1. Occludin

Occludin is highly expressed at cell-cell contact sites and is thought to be important in the assembly and maintenance of TJ. It consists of four transmembrane domains and two extracellular loops. The phosphorylation of occludin correlates with the regulation of its localization and function. Occludin that is phosphorylated at its serine/threonine residues is localized mainly in the membrane, whereas less phosphorylated occludin is found in the cytoplasm. In addition, phosphorylation of occludin appears to control its interaction with other TJ proteins such as ZO-1 [3]. The interaction between occludin and ZO-1 is essential for TJ integrity [4]. Therefore, phosphorylated occludin regulates TJ stability and permeability [5]. In keeping with these observations, occludin knockout mice showed normal TJ structure but exhibited elevated inflammation, hyperplasia, and growth retardation [6]. On the other hand, an enhanced level of occludin plays a role in further improving TJ barrier function and preventing damage to the TJ [7].

### 2.2. Claudin

Although there is no sequence homology between occludin and claudins, the claudin family also consists of four transmembrane domains and two extracellular loops that construct TJ strands (Figure 1). The claudin family is composed of 23 integral membrane proteins. The extracellular loops of claudin take part in heterophilic and hemophilic interactions with adjacent cells, which generate barriers against or pores for the passage of selective molecules in the paracellular pathways [8, 9]. It is thought that 27 mammalian claudin genes may exist, although their classification as claudins is disputed [10]. When the expression of claudins-1–24 was tested in rat and mouse intestines, all claudins except 6, 16, 19, 22, and 24 were detected by PCR analysis [11]. Although more studies are necessary to determine the exact functions of claudins in TJs, animal studies have indicated the importance of claudins in the integrity of TJs. Claudin-1-deficient mice exhibited abnormal TJ barrier formation, which induced cancer development and metastasis [12]. Mice with claudin-2 or claudin-15 deficiencies in the small intestine reveal that these transmembrane proteins play essential roles in the transepithelial paracellular channel-like permselectivity for extracellular monovalent cations, especially Na(+), in both infants and adults [13].

### 2.3. Zonula Occludens

The ZO proteins, which include ZO-1, ZO-2, and ZO-3, were the first TJ-specific proteins to be discovered [14]. ZO connects junctional proteins such as occludin and claudin to the actin cytoskeleton, and these protein interactions maintain TJ formation and function. To date, the roles of ZO proteins in TJs are not fully understood. Studies indicate that although ZO-1-deficient cells can maintain the structure of TJs and exhibit normal permeability, the activity of other TJ proteins such as occludin and claudins in assembling TJs was delayed in these cells [8, 15, 16]. On the other hand, the deficiency of ZO-2 or ZO-3 did not affect the formation of TJ in epithelial cell types [15], suggesting that ZO-1 proteins play a more important role in the control of TJ assembly compared to ZO-2 or ZO-3.

## 3. Disruption of Tight Junction

The integrity of the intestinal epithelial barrier maintained by TJ is crucial to protect the body against stress stimuli related to inflammation and infection. The alteration of TJ homeostasis is thought to induce the pathogenesis of several diseases and *vice versa*. Factors related to the alteration of TJ homeostasis include proinflammatory cytokines, pathogenic bacteria, lipopolysaccharides (LPS), and pathological conditions.

### 3.1. Proinflammatory Cytokines

Proinflammatory cytokines such as TNF-*α*, IL-1*β*, and IFN*γ* promote TJ permeability. TNF-*α* suppresses TJ barrier function due to the activation of the NF-*κ*B pathway and decreased ZO-1 protein level [17]. Conversely, blocking the NF-*κ*B pathway abolishes the TNF-*α*-mediated opening of the TJ barrier and ZO-1 downregulation. Interestingly, it appears that the TNF-*α* treatment site in filter-grown Caco-2 cell monolayers was important. TNF-*α* treatment in the basolateral compartment but not in the apical compartment significantly affected TJ integrity. The detailed molecular mechanism of the action of TNF-*α* was further studied *in vitro.* It has been proposed that an increase in MLCK expression and activity is associated with the NF-*κ*B-mediated disruption of TJ [18]. As the MLCK promoter region contains an NF-*κ*B binding site, TNF-*α* treatment increased MLCK promoter activity and transcription by NF-*κ*B activation [19]. Subsequently, enhanced levels of MLCK protein and its activity promoted TJ permeability in Caco-2 cells. Similarly, IL-1*β* increased TJ permeability via activation of the NF-*κ*B pathway [20]. MLCK activation by the extracellular signal-regulated kinases 1/2 (ERK1/2) signaling pathways was also related to IL-1*β* mediated changes in TJ [21, 22]. Unlike TNF-*α*, IL-1*β* treatment did not affect the ZO-1 protein level but did suppress the occludin protein level [20].

### 3.2. Pathogenic Bacteria and Lipopolysaccharides

The roles of pathogenic bacteria and bacterial toxins in the endothelial barrier have been reviewed elsewhere [23, 24]. Enteric pathogenic bacteria such as *Escherichia coli* (*E. coli*) and *Salmonella typhimurium* (*S. typhimurium*) alter the intestinal epithelial TJ barrier, leading to intestinal inflammation [24]. LPS, a component of the outer walls of gram-negative bacteria, is reported to alter TJ protein assembly, contributing to a leaky small intestine. Indeed, LPS is recognized by TLR4. TLR4 activation by LPS modulates the inflammatory response, which further exacerbates the alteration of TJ. *E. coli* O111: B4 LPS injection in mice disrupts intestinal epithelial TJ function in the ileal and colonic epithelia [25]. LPS also induces systemic inflammation, leading to altered expression and localization of TJ proteins such as ZO-1 and occludin. Apical LPS treatment increases TJ permeability and induces epithelial apoptosis by activating caspase-3 in duodenal epithelial monolayers [26]. Thus, it is thought that pathogenic bacteria and LPS disrupt intestinal TJ integrity by elevating various inflammatory signaling pathways, resulting in a leaky intestine.

### 3.3. Pathological Conditions

Certain pathological conditions are correlated with a defective intestinal TJ barrier, including inflammatory bowel disease (IBD), obesity, nonalcoholic steatohepatitis (NASH), and nonalcoholic fatty liver disease (NAFLD) (Table 1) [27].

#### 3.3.1. IBD

IBD involves a wide range of chronic remitting diseases of which ulcerative colitis (UC) and Crohn's disease (CD) are likely the most abundant [28]. IBD, usually known to involve a high level of intestinal inflammation, is associated with dysregulation of TJ [29]. IBD affects many components of the epithelial barrier including abnormal changes in the epithelium itself, its adhesion molecules, and the altered production of mucus and antimicrobial peptides. Together, these alterations result in the loss of solutes and fluid across the epithelial barrier, contributing to leak-flux diarrhea and elevated antigen translocation [28]. Antigen translocation in the *lamina propria* causes inflammation derived from circulating and resident immune cells, leading to further disruption of the barrier function [28, 30]. CD and UC are closely related to epithelial apoptosis [31], which is also a significant contributor to barrier leakiness. The apoptotic enterocytes invade the lumen while the remaining enterocytes redistribute the junctional proteins along the lateral cell membranes, leading to contraction of the surrounding epithelium and maintenance of barrier integrity [28, 32]. However, active UC is associated with the redistribution and decreased expression of claudin-1, claudin-4, claudin-7, and occludin, as well as a notable increase in claudin-2 expression [27, 33]. CD is also associated with both the redistribution and decreased expression of claudin-3, claudin-5, and claudin-8, as well as increased expression of claudin-2 [27, 33]. Furthermore, CD is known to present an abnormal intestinal structure with a high level of intestinal inflammation [34]. For example, patients with CD have elevated levels of plasma, fecal, and intestinal TNF-*α*s, which also can accelerate TJ dysfunction [35]. Altogether, the redistribution and alteration of TJ proteins, as well as inflammatory responses, are closely associated with barrier dysfunction in patients with UC or CD.

#### 3.3.2. Obesity

Obesity is associated with increased intestinal permeability. The impairment of intestinal barrier function due to the altered assembly of TJ proteins was observed in genetically obese mouse models including *ob/ob* and *db/db* mice [36]. The *ob/ob* and *db/db* mice exhibited an increase in intestinal permeability and significantly higher plasma endotoxin and proinflammatory cytokines such as IL-1*β*, IL-6, INF*γ*, and TNF-*α* compared to wild-type mice. TJ alteration has also been found in animal models with high-fat diet-induced obesity (DIO) and diabetes [37, 38]. High-fat diet caused suppression of the levels of occludin, claudin-1, claudin-3, and JAM-1, along with an increased level of plasma TNF-*α* in the small intestinal mucosa of rats [38]. DIO changed the gut bacterial population and TJ integrity in mice [37]. This study suggested that the microbiome is associated with DIO-induced endotoxemia and metabolic syndrome. These effects are possibly mediated by increased gut permeability followed by elevated LPS absorption. In support of this, antibiotics decreased circulating LPS levels, gut permeability, and metabolic syndrome [37]. Another study showed that the altered gut microbiota population in DIO mice is related to inflammation and gut permeability, partly due to the reduced expression of TJ-related genes including ZO-1 and occludin [39]. Involvement of LPS and TLR4 in DIO-induced TJ permeability has also been reported [40]. In the obese animals, MLC phosphorylation was significantly increased, causing disruption of TJ [40]. Antibiotics or prebiotics prevented the alteration of gut barrier function found in obesity and diabetes [37, 39]. These data indicate that obesity-induced inflammation is associated with changes in TJ integrity and gut microbiota.

#### 3.3.3. NASH and NAFLD

NASH and NAFLD, obesity-related fatty liver diseases, are also known to be associated with TJ dysfunction. The molecular mechanisms of these chronic liver diseases are not clear, but the interaction of microbiota, gut-liver axis, and obesity is emerging as a mechanism of the development of obesity-related liver disease [41]. Changes in the composition of gut microbiota in NAFLD increased circulating plasma LPS, subsequently triggering inflammation. These plasma LPS and proinflammatory cytokines concurrently increase intestinal permeability [42]. In patients with NAFLD, higher levels of insulin, blood pressure, serum triglycerides, total cholesterol, and liver enzymes were observed [43]. Patients with NAFLD also exhibited abnormal crypt and villi morphologies in duodenal mucosa, increased TJ permeability, and overgrowth of small intestinal bacteria. Increased intestinal permeability may cause the pathogenesis of hepatic fat deposition. In NAFLD, this increase in permeability mainly results from ZO-1 translocation in the crypt. Overall, inflammatory diseases such as inflammatory bowel disease, obesity, NASH, and NAFLD are highly associated with the disruption of TJ integrity.

## 4. Dietary Intervention to Maintain TJ Integrity

In order to develop a dietary intervention for TJ integrity, many factors in addition to efficacy should be considered, including safety, stability during processing and shelf life, the cost of developing raw ingredients, and sensory quality [44]. Regulation is also a factor to be considered, though food components are generally recognized as safe (GRAS). Given these criteria, bioactive food components are good candidate agents. Here, we focus on dietary food components to maintain TJ integrity.

### 4.1. Role of Phytochemicals in Tight Junction Integrity

**Figure 3:**
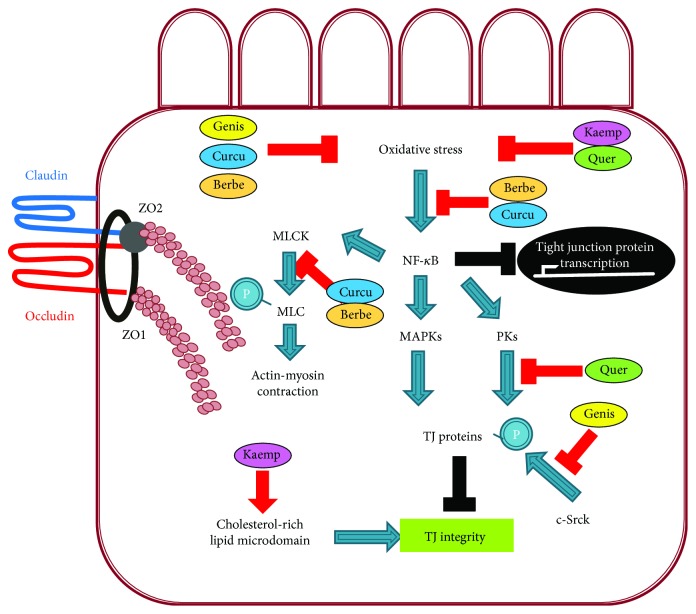
Hypothetical model of mechanisms underlying phytochemical-mediated protection of TJ integrity. MLCK: myosin light chain kinase; MAPK: mitogen-activated protein kinase; PKs: protein kinases (PKA, PKG, or PKC), c-SrcK: protooncogene tyrosine-protein kinase Src; Genis: genistein; Curcu: curcumin; Quer: quercetin; Berbe: berberine; Kaemp: kaempferol.

The beneficial role of dietary phytochemicals in TJ integrity has been reviewed previously [45, 46]. Of the many groups of phytochemicals, the effects of flavonoids (C_6_C_3_C_6_) on intestinal permeability have been widely studied. These secondary metabolites are widespread throughout the plant kingdom. Since the absorption rate of these phytochemicals is generally limited [45], it is likely that they reach both small and large intestines, affecting intestinal permeability. The effects of some of the most extensively studied flavonoids on the suppression of the paracellular permeability of epithelial cells that form TJs in the small intestine are summarized in Table 2. Next, we discuss the following phytochemicals: quercetin, berberine, genistein, kaempferol, and curcumin (Figure 3).

#### 4.1.1. Quercetin

Quercetin is one of the most widely distributed flavonoids in plants such as fruits, vegetables, and grains. Of its many biological activities, it is well-known for protecting cells from oxidative and inflammation-associated injuries. The biological functions of quercetin are closely associated with the regulation of key enzymes including PI-3 kinase, NF-*κ*B, PKC, tyrosine kinase, and the MAPK family [45, 47–49]; these enzymes (especially PKC and MAPKs) and their downstream signaling pathways are closely related to the assembly and integrity of TJ functions [45]. Thus, various studies have been undertaken to elucidate the roles of quercetin in TJ integrity. Quercetin augmented TJ barrier function in Caco-2 cells in the absence of any stimuli such as proinflammatory cytokines [50]. Treatment with 200 *μ*M quercetin for 24 hours specifically increased the expression of claudin-4 but not other TJ proteins such as occludin and claudin-1, -3, and -7. Another study showed that quercetin treatment elevated the transepithelial electrical resistance (TER) across the monolayers and reduced lucifer yellow flux, a paracellular marker [51]. In order to identify the cellular mechanisms involved in the beneficial effect of quercetin on TJ, several protein kinase inhibitors were used. Staurosporine, a general protein kinase inhibitor, and H7, an inhibitor of PKA and PKG, abrogated the preventive function of quercetin on TJ, indicating that the potential inhibition of PKA and PKG contributes to the protective effect on TJ by quercetin. Another study reported that 100 *μ*M quercetin reinforces TJ integrity through the modulation of multiple TJ-related proteins including claudin-1 and -4, ZO-2, and occludin by suppressing PKC*δ* [51]. Thus, it appears that the suppression of multiple protein kinases contributes to quercetin-mediated TJ integrity.

#### 4.1.2. Berberine

Berberine, found in *Coptidis rhizome*, is an isoquinoline alkaloid that has been used as a traditional Chinese medicine for thousands of years to treat gastrointestinal diseases, diarrhea, and bacterial infections [52], all of which are associated with the disruption of intestinal barrier function and TJ integrity. Consequently, the roles of berberine in intestinal TJ integrity have been studied both *in vivo* and *in vitro*. When the protective effect of berberine on intestinal damage was investigated in a mouse model of endotoxinemia, intragastric pretreatment with berberine partially prevented the ultrastructural damage of TJ partly by reversing the LPS-mediated redistribution of TJ proteins including occludin, ZO-1, claudin-1, or claudin-4 in colon epithelium and in membrane microdomains [53]. The protective effect of berberine on intestinal mucosal barrier dysfunction was also reported in type 2 diabetic rats [54]. Pretreatment with berberine for nine weeks significantly ameliorated the disruption of intestinal permeability, proinflammatory intestinal changes, and abnormal changes in gut-derived hormones [54]. *In vitro* studies also examined the beneficial effects of berberine on TJ integrity. Berberine at 100 *μ*M enhanced TJ in Caco-2 cells without any stimulation [55]. The combined treatment of Caco-2 cells with TNF-*α* and IFN*γ* induced TJ dysfunction through the cytosolic distribution of occludin; treatment with berberine prevented this dysfunction [56]. Although the mechanism underlying the berberine-mediated protection of TJ integrity requires further elucidation, studies indicate that the inhibition of the NF-*κ*B signaling pathway is involved in the beneficial effect of berberine on TJ. The NF-*κ*B signaling pathway is central in stimulating the transcription of diverse inflammatory genes. It has been reported that the intestinal NF-*κ*B p65 subunit is activated in the endotoxinemic state, but berberine treatment reduced these effects [53]. In addition, berberine supplementation suppressed the NF-*κ*B signaling pathway in the intestine of diabetic rats [54]. The berberine-mediated inhibition of NF-*κ*B signaling is partially due to the suppression of inhibitory factor *κ*B (I-*κ*B) kinase, which stabilizes I-*κ*B, thereby inhibiting nuclear translocation of the NF-*κ*B p65 subunit [53].

#### 4.1.3. Genistein

Genistein is a major isoflavone present in soybeans. Since it is well-known as a potent inhibitor of protein tyrosine kinases, many studies have focused on the effects of genistein on signal transduction [45]. As mentioned earlier, the phosphorylation status of TJ proteins is highly associated with TJ function and structure [57, 58]. Thus, various studies have reported the roles of genistein in TJ integrity. Genistein tightened TJ in Caco-2 cells [59]. When enteric bacteria such as *E. coli* and *S. typhimurium* interacted with intestinal cells, TJ barriers were opened, but genistein at 300 *μ*M prevented this opening and blocked the invasion of enteric bacteria. Another study showed that genistein improved intestinal TJ barrier dysfunction induced by oxidative stress [60]. Treatment with a mixture of xanthine oxidase and xanthine, which induces oxidative stress, reduced the TER and elevated [^3^H]-mannitol flux, both of which represent TJ permeability; the coadministration of genistein at 300 *μ*M inhibited these changes partially by suppressing oxidative stress-induced c-Src kinase, followed by the inhibition of the tyrosine phosphorylation of TJ proteins [60]. The protective effects of genistein on TJ permeability are partly ascribed to the inhibitory effect on the phosphorylation of TJ proteins. Occludin undergoes tyrosine phosphorylation when TJ function is impaired by various factors [61], but genistein appears to suppress this process [45]. Genistein also ameliorates oxidative stress-induced TJ barrier dysfunction. Oxidative stress induced by the mixture of xanthine oxidase and xanthine, which produces superoxide anions in culture media, reduced TER and elevated [^3^H]-mannitol flux, markers of TJ permeability in Caco-2 cells; these alterations were reversed by the coadministration of genistein [60]. As an underlying mechanism, genistein appears to inhibit oxidative stress-stimulated c-Src kinase activation, preventing the tyrosine phosphorylation of TJ proteins (ZO-1 and occludin) and adherence junction (AJ) proteins (E-cadherin). These actions suppress the disassembly of TJ and AJ proteins from the junctional complex [45, 60].

#### 4.1.4. Kaempferol

Kaempferol is a flavonol found in fruits and vegetables including apples, grapes, broccoli, kale, and chives. Numerous *in vivo* and *in vitro* studies have demonstrated that kaempferol and kaempferol glycosides exhibit health-promoting effects including antioxidant and anti-inflammatory benefits, which are associated with the maintenance of TJ integrity [62]. When the roles of kaempferol in TJ functions were investigated, it was found to strengthen the TJ barrier in Caco-2 cells [63]. Kaempferol notably elevated TER across the monolayers. The administration of kaempferol at 100 *μ*M promoted the protein expression of TJ-related proteins ZO-1 and -2, occludin, and claudin-1, -3, and -4 and elevated the phosphorylation of occludin. Consistently, microscopic analysis indicated that kaempferol stimulated the assembly of occludin and claudin-3 at TJs [63]. As an underlying mechanism, the membrane lipid microdomain is associated with the kaempferol-mediated beneficial effects on TJ as evidenced by the inhibition of kaempferol-induced elevation of TER after extraction of cholesterol with methyl-*β-*cyclodextrin and kaempferol-mediated increase in the TJ protein distributions in the cholesterol-rich lipid microdomain [63]. These data suggest that the membrane lipid microdomain is closely related to the increase in TJ protein assembly and intestinal TJ integrity by kaempferol [63].

#### 4.1.5. Curcumin

Curcumin is a biologically active polyphenolic compound found in the dietary spice turmeric. It has long been used in Asian countries as a remedy for various diseases including diabetes, liver diseases, infectious diseases, and cancers [64]. It has been shown to suppress chronic inflammatory diseases despite poor bioavailability [65]. Thus, it has been hypothesized that curcumin may act on intestinal epithelial cells to regulate systemic inflammation [66]. Consequently, a potential role for curcumin in protecting TJ integrity has been proposed. When Caco-2 cells were stimulated by the proinflammatory cytokines TNF-*α* or IL-1*β*, epithelial TJ permeability was significantly increased through the activation of the NF-*κ*B pathway. Curcumin pretreatment abolished proinflammatory cytokine-deteriorated TJ barrier function. Moreover, curcumin treatment prevents apical leptin-impaired TJ by suppressing the leptin signaling pathway in Caco-2 BBe monolayers [67]. Curcumin suppressed the gene expression of leptin-induced proinflammatory cytokines such as IL-6 and TNF-*α* and leptin-induced genes such as *c-fos* and *c-jun*. Moreover, curcumin blocked leptin-altered TJ gene expression including ZO-1, claudin-5, and occludin. Another study using intestinal epithelial cells showed that pretreatment with curcumin ameliorated the LPS-mediated disruption of intestinal barrier function [66]. Although the underlying mechanisms must be further investigated, various studies used curcumin as an inhibitor of the NF-*κ*B pathway in proinflammatory cytokine-induced TJ alteration [17, 19, 20]. It suppresses NF-*κ*B activity by inhibiting I-*κ*B kinase followed by the stabilization of I-*κ*B [68, 69]. Another mechanism underlying the curcumin-mediated protective effect on TJ includes the inhibition of the IL-1*β*/p38 signaling cascade. IL-1*β*-mediated activation of p38 MAP kinase results in the activation of MLCK. MLCK-mediated phosphorylation of myosin light chain impairs TJ structure and elevates intestinal permeability [66].

## 5. Conclusions

TJ is associated with physical intestinal barrier function, regulating the paracellular movement of various substances across the intestinal epithelium. Studies reveal that TJ dysfunction is closely related to inflammatory and metabolic disorders including IBD, NASH, NAFLD, and obesity via the disruption of TJ barrier functions. Thus, the maintenance of TJ integrity is likely a good strategy to prevent and/or treat these diseases. Although natural compounds such as quercetin, berberine, genistein, kaempferol, and curcumin have been reported to improve TJ integrity by controlling TJ-related proteins and inflammatory signaling pathways (Figure 3), more detailed molecular studies on the effects of natural compounds on intestinal TJ functions are necessary to develop preventive medicine and pharmaceutical agents against inflammatory and metabolic diseases.

## Figures and Tables

**Table 1:** Potential diseases associated with the disruption of tight junction.

Diseases	Reported intestinal symptom	References
Inflammatory bowel disease	Dysfunction of the intestinal barrier, chronic inflammation	
Crohn's disease	An abnormal intestinal structure, a leaky small intestine, a high level of intestinal inflammation	[34]
Obesity	The impairment of intestinal barrier function, alteration in microbiome population	[37]
NAFLD	Abnormal morphologies of crypts and villi in duodenal mucosa	[43]
Coeliac disease (autoimmune enteropathy)	Dysfunction of the intestinal barrier (increased gliadin permeation and related immune response)	
Type 1 diabetes mellitus	Gut microbiota dysbiosis, increased intestinal permeability, heightened immune activation	

**Table 2:** The effects of phytochemicals on the tight junction barrier function.

Treatment	Conc.	System	Stimuli	Tight junction protein affected	Potential targeting pathways	Ref.
Quercetin	200 *μ*M	Caco-2	None	↑ claudin-4	↓ protein kinase A (PKA) or PKG	[50]
	100 *μ*M	Caco-2	None	↑ ZO-2, occludin, claudin-1 and -4	↓ PKC*δ*	[51]

Berberine	100 *μ*M	Caco-2	None	Not studied	Not studied	[55]
	100 *μ*M	Caco-2	TNF-*α*, IFN*γ*	↑ occludin	Not studied	[56]
	200 mg/kg	Mouse	LPS	↑ occludin, ZO-1, claudin-1 and -4	↓ myosin light chain kinase, ↓ nuclear factor-*κ*B (NF-*κ*B)	[53]
	375 mg/kg/day	Rat	Streptozotocin	↑ occludin, ZO-1, claudin-1	↓ TLR4/MyD88/NF-*κ*B signaling pathways	[54]

Genistein	300 *μ*M	Caco-2	None	Not studied	Localization of filamentous actin in the perijunctional area	[59]

Kaempferol	100 *μ*M	Caco-2	None	↑ occludin, claudin-1 and -3, and ZO-1; ↑ ZO-2 and claudin-4; phosphorylation of occludin	Not studied	[63]

Curcumin	5 *μ*M	Caco-2	TNF-*α*	↑ ZO-1	↓ NF-*κ*B	[17]
	5 *μ*M	Caco-2	TNF-*α*	Not studied	↓ myosin light chain kinase, ↓ NF-*κ*B	[19]
	5 *μ*M	Caco-2	IL-1*β*	Not studied	↓ NF-*κ*B	[20]
	30 *μ*M	Caco-2 BBe	Leptin	↑ ZO-3, claudin-5, occludin	↓ leptin signaling pathway	[67]
